# Coronary patients with high plasma omentin are at a higher cardiovascular risk

**DOI:** 10.1016/j.dib.2015.11.065

**Published:** 2015-12-11

**Authors:** Christoph H. Saely, Andreas Leiherer, Axel Muendlein, Alexander Vonbank, Philipp Rein, Kathrin Geiger, Cornelia Malin, Heinz Drexel

**Affiliations:** a Department of Medicine and Cardiology, Academic Teaching Hospital Feldkirch, Feldkirch, Austria; b Vorarlberg Institute for Vascular Investigation and Treatment (VIVIT), Feldkirch, Austria; c Private University of the Principality of Liechtenstein, Triesen, Liechtenstein; d Drexel College University of Medicine, Philadelphia, PA, USA

**Keywords:** Omentin, Adipokine, Adipose tissue, Atherosclerosis, Coronary artery disease, Coronary angiography, Prognostic factor, Prospective cohort study

## Abstract

The adipokine omentin, also known as intelectin, is a secretory protein, expressed in visceral adipose tissue and is highly abundant in plasma. It is involved in the development of chronic inflammatory diseases, but nothing is known about its impact on the cardiovascular event risk. Here, plasma omentin was measured in 295 patients undergoing coronary angiography for the evaluation of established or suspected stable coronary artery disease (CAD). Patients were separated according to the median plasma omentin concentrations into a high and low omentin group and cardiovascular events occurring during a period of 3.5 years have been recorded. We observed that patients within the high omentin group had significantly more cardiovascular events than patients in the low omentin group. This was true even if using different study endpoints. This article describes data related to a research article titled “High Plasma Omentin Predicts Cardiovascular Events Independently From the Presence and Extent of Angiographically Determined Atherosclerosis” (Saely et al., 2015) [1].

Specifications Table

**Table:** 

Subject area	*Clinical Research*
More specific subject area	*Epidemiology, Biomarkers*
Type of data	*Figure*
How data was acquired	*Prospective study*
Data format	*Analyze data*
Experimental factors	*Omentin in plasma sample was measured by ELISA*
Experimental features	*Omentin concentration in 295 coronary patients has been determined and cardiovascular events have been recorded during3.5 years.*
Data source location	*Feldkirch, Austria*
Data accessibility	*Data is with this article*

## 1 Value of the data

– Up to date, no prospective data on the power of the adipokine omentin to predict cardiovascular events are available.
– The data from our prospective cohort study for the first time shows that elevated plasma omentin significantly predicts cardiovascular events.
– This data are important, because the limited existing literature on omentin had rather suggested benefits on cardiometabolic parameters in other populations.
– The adipokine omentin may serve as a potential new biomarker in coronary patients.
– The data may stimulate future research addressing omentin in different patient cohorts.

## 2 Data

**Fig. 1:**
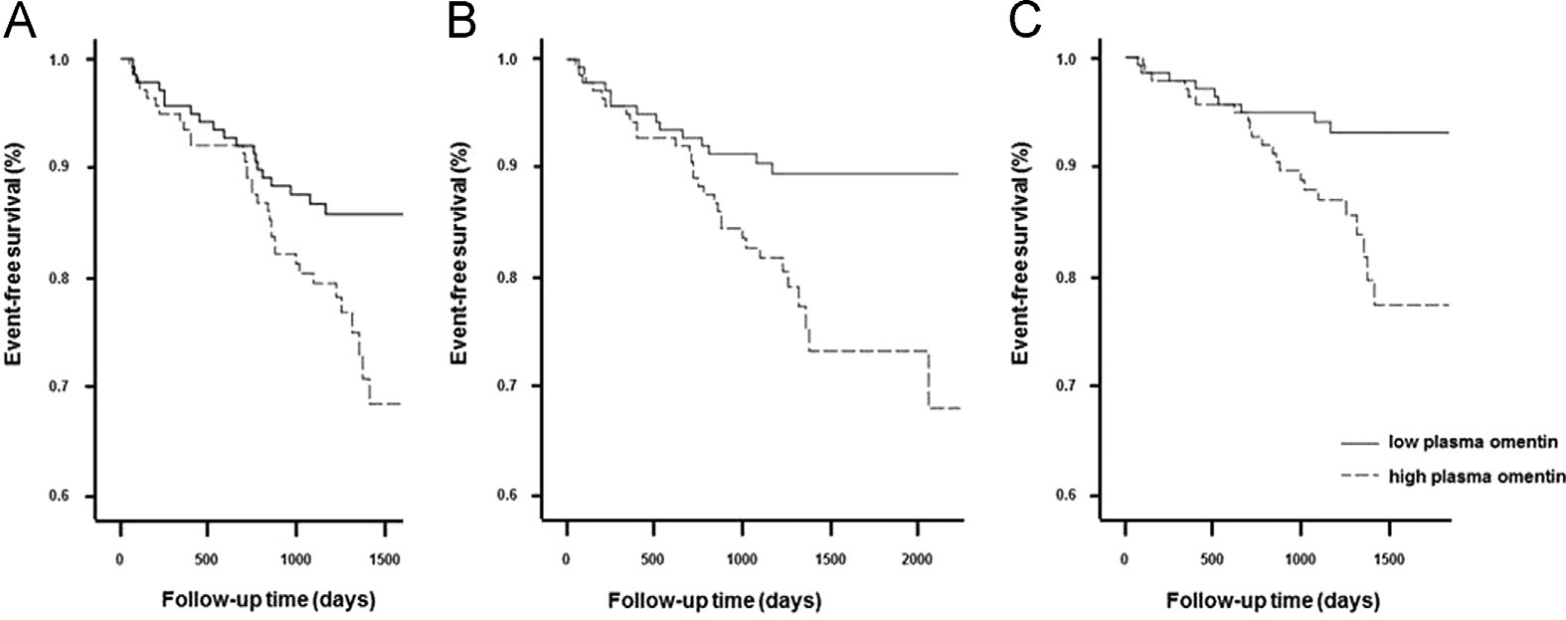
Incidence of study endpoints by high and low omentin concentration in coronary patients. The survival plot indicates event-free survival according to low (4.0–13.44 ng/ml) and high plasma omentin concentration (13.45–108.3 ng/ml) in angiographied patients during 3.5 years. Study endpoint (EP) A comprise all cardiovascular events, including coronary death, fatal ischemic stroke, non-fatal myocardial infarction, non-fatal ischemic stroke, and need for coronary artery bypass grafting, percutaneous coronary intervention, or revascularization in the carotid or peripheral arterial beds (A). EP B comprises all events of EP A but without revascularization in the carotid or peripheral arterial beds (B). EP C comprises only major cardiovascular events, including coronary death, fatal ischemic stroke, non-fatal myocardial infarction, and non-fatal ischemic stroke (C).

The data presented here show the incidence of cardiovascular events and study endpoints (EP) respectively in 295 coronary patients according to their plasma omentin concentration. Patients were separated into a group with high (above the median, *n*=147) and low (below the median, *n*=148) omentin. The event-free survival (Fig. 1) was significantly higher in the low omentin group than in the high omentin group if including all cardiovascular events (EP A, _pLog-Rank_=0.037), if including all cardiovascular events but excluding revascularization in arterial beds (EP B p_Log-Rank_=0.012), or if including only major cardiovascular events (EP C; _pLog-Rank_=0.019).

## 3 Experimental design, materials and methods

### 3.1 Patients

From a large cohort of 1751 Caucasian patients, who were referred to coronary angiography for the evaluation of established or suspected stable coronary artery disease (CAD), 295 were randomly drawn, except for CAD status (161 had significant CAD and 134 did not have significant CAD) and sex (140 women and 155 men). Patients with acute coronary syndromes were not included. In these 295 patients, we measured plasma omentin. Subsequently, these 295 patients were separated according to the median of measured omentin concentrations into a high omentin group (*n*=147) and a low omentin group (*n*=148). The present study has been approved by the Ethics Committee of the University of Innsbruck; written informed consent was given by all participants.

## 4 Laboratory analyses

Venous blood samples were collected after an overnight fast of 12 h before angiography was performed and laboratory measurements were performed from fresh plasma samples. Plasma omentin levels were determined with a commercial omentin enzyme-linked immunosorbent assay (ELISA) kit (catalog no. SK00010-01; Aviscera Bioscience, CA, USA) from the same batch of kits (lot no. 20110234), specific for omentin-1 with an inter-assay variation less than 10%. We assessed a mean (+SD) omentin concentration of 17.4 (+14.3) ng/ml and a median of 13.4 (interquartile range 7.9–21.5) ng/ml. Thus the data range is comparable to omentin levels reported for healthy control subjects ranging from 14 to 34 ng/ml [2], [3] and is also in line with other studies [3], [4], [5], [6], [7], [8].

## 5 Prospective study

Mortality data were collected annually from a national survey (Statistik Austria, Vienna, Austria); follow-up visits to our institution were planned after 3–4 years in order to assess endpoints among survivors. The mean follow up time was 3.5 ±1.1 years after the baseline investigation. To assess non-fatal endpoints we conducted standardized interviews; additionally, hospital records were reviewed.

The data refer to cardiovascular events comprising coronary death (fatal myocardial infarction, sudden cardiac death, mortality from congestive heart failure due to CAD), fatal ischemic stroke, non-fatal myocardial infarction non-fatal ischemic stroke, and need for coronary artery bypass grafting (CABG), percutaneous coronary intervention (PCI), or revascularization in the carotid or peripheral arterial beds. The combination of all these events has been referred to as our primary study endpoint (EP A). Additional endpoints include the composite of coronary death (fatal myocardial infarction, sudden cardiac death, mortality from congestive heart failure due to CAD), fatal ischemic stroke, non-fatal myocardial infarction, non-fatal ischemic stroke, and need for coronary artery bypass grafting (CABG) or percutaneous coronary intervention (PCI) – i.e. the primary endpoint without revascularization in the carotid or peripheral arterial beds (=EP B); and the composite of coronary death (fatal myocardial infarction, sudden cardiac death, mortality from congestive heart failure due to CAD), fatal ischemic stroke, non-fatal myocardial infarction and non-fatal ischemic stroke – i.e. major cardiovascular events (=EP C).

Coronary angioplasty and bypass surgery were considered as cardiovascular events unless they were planned as a consequence of the baseline angiography and therefore were not “future” events. A follow-up rate of 95% was achieved.

## 6 Statistical analysis

Survival analysis was performed using Kaplan–Meier and Log-Rank tests respectively. Patients who were lost during the follow-up (*n*=15) were not included in the prospective analyses. Statistical analyses were performed with the software package SPSS 22.0 for Macintosh, IBM SPSS; Chicago, IL.

## References

[1] Saely C.H., Leiherer A., Muendlein A., Vonbank A., Rein P., Geiger K., Malin C., Drexel H. (2015). High plasma omentin predicts cardiovascular events independently from the presence and extent of angiographically determined atherosclerosis. Atherosclerosis.

[2] Li X.P., Zeng S., Wang M., Wu X.P., Liao E.Y. (2014). Relationships between serum omentin-1, body fat mass and bone mineral density in healthy Chinese male adults in Changsha area. J. Endocrinol. Invest..

[3] Guenes M., Bukan N. (2015). Examination of angiopoietin-like protein 4, neuropeptide Y, omentin-1 levels of obese and non-obese patients with polycystic ovary syndrome. Gynecol. Endocrinol..

[4] Yin J., Hou P., Wu Z., Nie Y. (2015). Decreased levels of serum omentin-1 in patients with inflammatory bowel disease. Med. Sci. Monit..

[5] Yan P., Liu D., Long M., Ren Y., Pang J., Li R. (2011). Changes of serum omentin levels and relationship between omentin and adiponectin concentrations in type 2 diabetes mellitus. Exp. Clin. Endocrinol. Diabetes.

[6] Kadoglou N.P., Tahmatzidis D.K., Giannakoulas C., Kapelouzou A., Gkontopoulos A., Parissis J., Lampropoulos S., Kottas G. (2015). Serum levels of novel adipokines, omentin-1 and chemerin, in patients with acute myocardial infarction: KOZANI STUDY. J. Cardiovasc. Med..

[7] Li X.P., Zeng S., Wang M., Wu X.P., Liao E.Y. (2014). Relationships between serum omentin-1, body fat mass and bone mineral density in healthy Chinese male adults in Changsha area. J. Endocrinol. Invest..

[8] Tan B.K., Adya R., Farhatullah S., Chen J., Lehnert H., Randeva H.S. (2010). Metformin treatment may increase omentin-1 levels in women with polycystic ovary syndrome. Diabetes.

